# Very Bright Green Fluorescent Proteins from the Pontellid Copepod *Pontella mimocerami*

**DOI:** 10.1371/journal.pone.0011517

**Published:** 2010-07-14

**Authors:** Marguerite E. Hunt, Michael P. Scherrer, Frank D. Ferrari, Mikhail V. Matz

**Affiliations:** 1 Institute for Cellular and Molecular Biology, The University of Texas at Austin, Austin, Texas, United States of America; 2 Department of Invertebrate Zoology, National Museum of Natural History, Smithsonian Institution, Suitland, Maryland, United States of America; 3 Section of Integrative Biology, The University of Texas at Austin, Austin, Texas, United States of America; Cairo University, Egypt

## Abstract

**Background:**

Fluorescent proteins (FP) homologous to the green fluorescent protein (GFP) from the jellyfish *Aequorea victoria* have revolutionized biomedical research due to their usefulness as genetically encoded fluorescent labels. Fluorescent proteins from copepods are particularly promising due to their high brightness and rapid fluorescence development.

**Results:**

Here we report two novel FPs from *Pontella mimocerami* (Copepoda, Calanoida, Pontellidae), which were identified via fluorescence screening of a bacterial cDNA expression library prepared from the whole-body total RNA of the animal. The proteins are very similar in sequence and spectroscopic properties. They possess high molar extinction coefficients (79,000 M^−1^ cm^−^) and quantum yields (0.92), which make them more than two-fold brighter than the most common FP marker, EGFP. Both proteins form oligomers, which we were able to counteract to some extent by mutagenesis of the N-terminal region; however, this particular modification resulted in substantial drop in brightness.

**Conclusions:**

The spectroscopic characteristics of the two *P. mimocerami* proteins place them among the brightest green FPs ever described. These proteins may therefore become valuable additions to the *in vivo* imaging toolkit.

## Introduction

Since first being isolated from the bioluminescent jellyfish *Aequorea victoria*, phylum Cnidaria, green fluorescent protein (GFP) and its derivatives have accelerated life science research by being extensively used as genetically encoded *in vivo* markers [1], [2], [3], [4], [5]. Past rationale suggested that fluorescent proteins would be exclusively found in cnidarians and that these proteins would also necessarily be coupled to the luminescent systems that are common in these marine animals. However, this view changed with the discovery of GFP-like proteins in non-luminescent organisms such as corals (Phylum Cnidaria, class Anthozoa), as well as representatives of other phyla: copepods (phylum Arthropoda, class Crustacea), and amphioxus (phylum Chordata, subphylum Cephalochordata) [6], [7], [8], [9], [10], [11], [12].

Seven GFP-like proteins have been identified thus far from the copepod families Pontellidae and Aetideidae [10], [13]. In general, the GFP-like proteins from this group of animals have qualities such as rapid florescence development following protein synthesis, high brightness, and increased photostability, all extremely valuable for use as in biotechnology tool. Isolation and characterization of more GFP-like proteins in copepods will likely continue to provide better fluorescent proteins for use in biomedical research.

In this study, we cloned, expressed and characterized two GFP-like proteins from a Pontellid copepod *Pontella mimocerami* Fleminger, 1957 [14], collected in the Bahamas. The two proteins are very similar to each other in their amino acid sequences and spectral characteristics, closely resembling other copepod GFP-like proteins; however, their brightness characteristics (molar extinction coefficient and quantum yield of fluorescence) suggest that they are among the brightest green fluorescent proteins described thus far.

## Materials and Methods

### *Pontella mimocerami* collection and total RNA isolation

**Figure 1:**
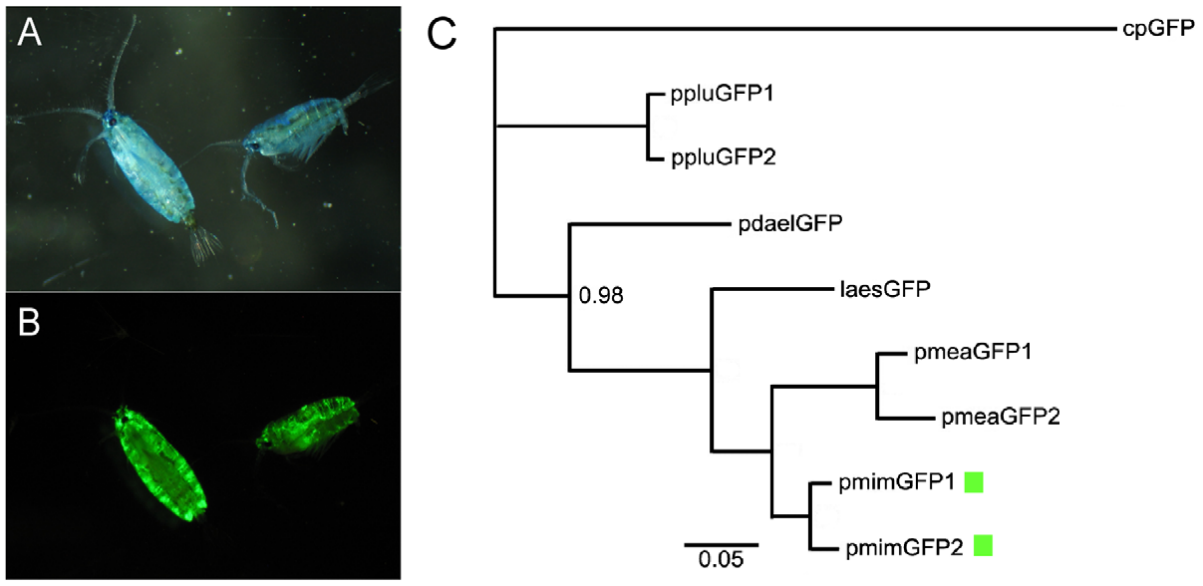
Source organism and phylogenetic relationships of the new proteins. A: *Pontella mimocerami* illuminated and imaged under white light. Note the blue non-fluorescent coloration, which is typical of many pontellid species. B: Same specimens under blue light showing the bright green fluorescence. On panels A and B, female is on the left, male is on the right. C: Bayesian phylogeny of the protein-coding sequences of all known GFPs from copepods of the Pontellidae family, rooted with cpGFP from *Chiridius poppei*, a copepod from the family Aetideidae. Scale bar: 0.05 substitutions per nucleotide site. The posterior probability at each node is 1.00 except where indicated. The two new proteins are indicated by green rectangles.

The copepods were collected during a sunset plankton tow at 8pm on August 19, from the stern of the RV Seward Johnson during the 2007 Deep Scope Cruise. The samples were collected at 25°1.3′N, 77°36.2′W by towing a 200 µm plankton net at 5–15 ft below the surface at 1 knot for 20 minutes. The collected organisms were inspected with a blue flashlight (BlueStar, NightSea; Andover, MA). Several bright green fluorescent copepods were thus caught, and identified to the family level, Pontellidae. The organisms were photographed under white and blue light under MZ FL III stereomicroscope (Leica; Bannockburn, IL), equipped with Powershot G6 camera (Canon; Lake Success, NY), using filter set #11003 BL/VIO (Chroma Technology Corp; Rockingham, VT, Fig. 1A, B). Total RNA was extracted using RNAqueous kit (Ambion, Austin, TX) according to manufacturer's protocol and stored in 6.65 M LiCl at −80°C. The specimens for identification were preserved by freezing in Tissue-Tek O.C.T. compound and stored at −80°C (Sakura Finetek; Torrance, CA).

### Preparation and screening of bacterial cDNA expression library

cDNA was synthesized and PCR-amplified using SMART cDNA amplification kit (Clontech; Mountain View, CA) and SuperScript II reverse transcriptase (Invitrogen; Carlsbad, CA), with two modifications. First, a different oligonucleotide was used for priming the reverse transcription reaction: 5′AAGCAGTGGTATCAACGCAGAGTCGCAGTCGGTAC(T)_13_V (where V stands for a mixture of A, G, and C bases). For the first step in cDNA amplification, the following long oligonucleotide was used in lieu of the one provided with the SMART cDNA amplification kit: AGT GGA CTA TCC ATG AAC GCA AAG CAG TGG TAT CAA CGC AGA GT 3′. The PCR reactions contained 0. 3 µM of the primer. The thermocycler profile was: 94°C for 5 m, 94°C for 40 s, 68°C for 4 m, cycle to step two for 26 additional cycles, hold at room temperature. The product from this step was diluted 1∶10 and 3 µl of this dilution was used for the second step in cDNA amplification. For this second amplification step, three separate reactions were performed. The first one used the same oligonucleotide as in the first amplification step; the other two reactions used the same oligonucleotide, but extended by either one or two T bases at the 5′ terminus. These PCR reactions contained 0. 1 µM of the primer, the thermocycler profile was 94°C for 5 m, 94°C for 40 s, 68°C for 4 m, cycle to step two for 5 additional cycles, hold at room temperature. Such conditions bias the PCR amplification towards longer products [15], generating a cDNA sample enriched with full-length coding regions. The second amplification ensured that, upon ligation into vector, each cDNA species would be represented by inserts fused to the leading lacZ peptide in all three possible reading frames. The products of amplification were purified using QIAquick PCR Purification Kit (Qiagen; Valencia, CA) and ligated into pGEM-T vector (Promega; Madison, WI) following manufacturers' protocols. The ligations were transformed into TOP 10 chemically competent *Escherichia coli* cells (Stratagene; Cedar Creek, TX) and the resulting library was plated onto Luria Bertani (LB)/Agar plates supplemented with 100 µg/ml ampicillin and 1 mM Isopropyl β-D-1-thiogalactopyranoside (IPTG). The plates were incubated overnight at 37°C and then screened at one day post-transformation for green fluorescent colonies using a Leica MZ FLIII microscope with GFP specific filter # 51004v2 F/R (Chroma Technology Corp). A total of about 10^5^ bacterial colonies were surveyed.

### Identification, expression, and purification of *Pontella mimocerami* GFPs

We identified six green fluorescent colonies, which were picked into individual 3 ml LB/Amp (100 µg/ml final Amp concentration) bacterial cultures and grown overnight at 37°C. The cultures were processed using QIAprep Spin Miniprep Kit (Qiagen) following the manufacturer's protocol. 500 ng of each of the six plasmids were sequenced using an ABI 3730 sequencer (Applied Biosystems; Foster City, CA). The sequences were aligned using SeqMan2 software (DNASTAR Lasergene 7.2; Madison, WI) and gene identity was confirmed by BLASTX [16] searching a non-redundant protein database. From the sequences two GFP-like isoforms were identified. We chose two plasmid constructs, pmimGFP1 (Genbank accession number GQ247522) and pmimGFP2 (Genbank Accession number GQ247523), representing each isoform, to use as templates to re-amplify the gene coding regions from the representative plasmids. The upstream primer had a 5′-heel comprising 3 leading stop codons followed by a Shine-Dalgarno sequence [17], 6-base linker, and initiation codon (5′-TTG ATT GAT TGA AGG AGA AAT ATC ATG, [18]), and the downstream primer had a 5′-heel with a 6-histidine tag encoded in front of the stop codon (reverse complement of 5′-CAT CAC CAT CAC CAT CAC TAA A, [18]). The resulting amplicons were ligated into pGEM-T vector (Promega) and transformed into Z strain of *E.coli* (Zymo Research; Orange, CA), which in our experience was optimal for heterologous expression of FPs. The transformations were plated onto LB/Agar plates supplemented with 1x Amp and 1x IPTG (concentrations as previously noted), and incubated overnight at 37°C. One green fluorescent colony was picked from each plate, suspended in 20 µL of water, and streaked onto fresh LB/Agar plates supplemented with 100 µg/ml ampicillin and 1 mM IPTG. After a two-day incubation at room temperature, the colonies were harvested from plates and suspended in 1xPBS, sonicated on ice, and centrifuged to remove the cellular debris. We used the cleared lysate to isolate a purified solution of the green fluorescent protein using metal-affinity chromatography as implemented in QIA-Expressionist system following the manufacturer's protocol (Qiagen). The fluorescent proteins were eluted in 500 mM imidazole in 1xPBS. The imidazole was removed by buffer exchange for 1xPBS by repeated centrifugation steps in a protein concentrator (Amicon Ultra – 15, Millipore; Billerica, MA). The resulting protein concentration was measured using BCA method (Pierce; Rockford, IL).

### Phylogenetic analysis

A nucleotide alignment of all copepod GFP-like proteins was prepared with Geneious software v 3.7 (19). The Bayesian phylogenetic analysis (Fig. 1C) was performed on the basis of coding nucleotide sequence alignment, using MrBayes software embedded within Geneious package [19], [20]. The analysis was based upon a generalized time reversible model (GTR, [21]) and involved 1,100,000 Markov chain Monte Carlo, (MCMC) steps. The trees were sampled every 200 steps generating 5,500 trees, of which the first 5,000 were discarded (“burned”), and the remaining 500 trees were used to infer posterior probabilities. The tree was rooted by the closely related cpGFP from *Chiridius poppei*, which comes from another family of calanoid copepods (Aetideidae, [13]). An amino acid sequence alignment, including *A. victoria* GFP, was prepared with Geneious software [19]. The analyzed sequences included GFPs from *Chiridius poppei* (cpGFP, Accession No. AB185173), *Labidocera aestiva* (laesGFP, No. AY268073), unidentified *Pontella* (pdaelGFP, AY268076), *Pontella meadi* (pmeaGFP1 and GFP2, Nos. AY268074 and AY268075), and *Pontella plumata* (ppluGFP1 and GFP2, Nos. AY268071 and AY268072).

### Spectroscopy

**Figure 2:**
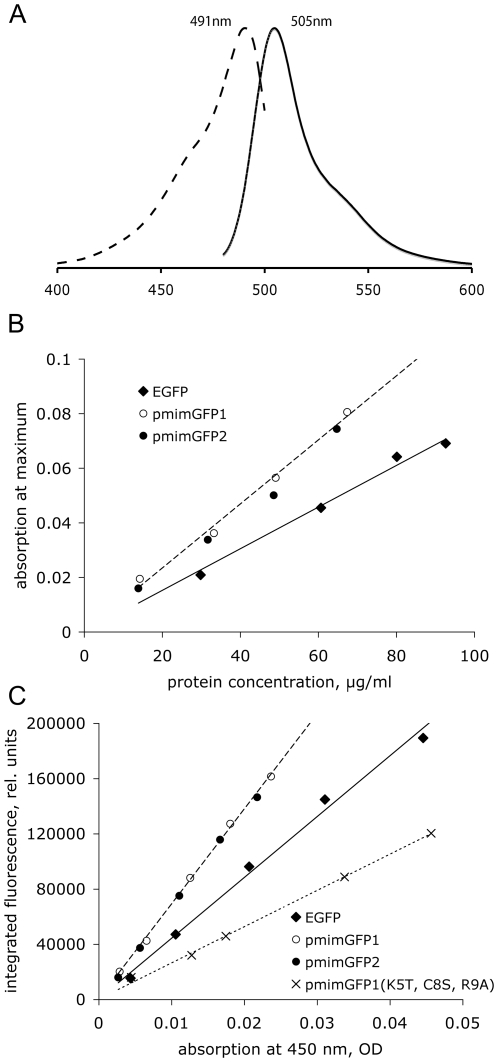
Spectroscopic properties of the new proteins. A: Normalized excitation (dashed line) and emission (solid line) curves of purified pmimGFP1 and pmimGFP2 (the curves for these two proteins are identical). Horizontal axis: wavelength in nanometers; vertical axis: fluorescence amplitude. B: Comparison of extinction, at each protein's own absorption maximum, between EGFP and the new proteins. C: Comparison of quantum yields of fluorescence (QY) between EGFP, new proteins, and the non-aggregating mutant of pmimGFP1.

The excitation and emission spectra of the bacterial expression products were measured using LS-50B spectrofluorometer (Perkin Elmer; Waltam, CT), and corrected for the photomultiplier sensitivity. The brightness characteristics (molar extinction coefficient, ME, and quantum yield of fluorescence, QY) of the new proteins were evaluated in direct comparison to the most widely used FP marker, EGFP [22]. A range of protein dilutions was prepared in 1x PBS supplemented with 250 mM imidazole (to ensure solubility of the new proteins), both for the standard (EGFP, BioVision, Montain View, CA) and *P. mimocerami* proteins. These dilutions were evaluated for absorption (400–550 nm), fluorescence (480–700 nm, excited at 450 nm), and protein concentration according to the BCA assay (Pierce), in identical conditions within the same microtiter plate, using SpectraMax M2 microplate reader with the provided software (Molecular Devices, SoftMax Pro v5; Sunnyvale, CA). The dilution factors were selected to achieve absorption at excitation within 0.01–0.05 OD range, both for standard and unknowns, to minimize secondary absorption-emission that could distort QY measurements. The ME and QY were calculated relative to their known values of EGFP (ME = 55,000 M^−1^ cm^−1^, QY = 0.6, [22]), from the difference in the slopes of linear regressions of absorption at maximum versus protein concentration for ME (Fig. 2B), and of integrated total fluorescence versus absorption for QY (Fig. 2C).

### Oligomerization and aggregation

To determine the oligomeric status of our new copepod GFP-like proteins, we analyzed the proteins using SDS-PAGE in a 4–15% gradient gel with SDS-Tris-Glycine buffers (Bio-Rad, Hercules, CA). To resolve GFP-like proteins in the native state, the samples were not boiled before loading on the gel, and visualized after the run by their native fluorescence. This method of oligomerization assessment utilizes the fact that most GFP-like proteins do not lose their oligomeric state or fluorescence unless boiled in SDS, and their mobility in the gel correlates with their globular size. This was first noticed and exploited in studies of oligomerization of the red fluorescent protein DsRed [23], followed by demonstration of the utility of this approach for assessing oligomerization in a variety of other GFP-like proteins [24], [25]. Since it is theoretically possible that SDS would disrupt oligomers but not unfold the protein, the method is applied conservatively, such that the only result that is considered relevant is the presence of oligomerization or aggregation, whereas the apparent lack of oligomerization does not necessarily imply the monomeric state of the protein in the absence of SDS. Since the mobility of such non-denatured protein does not correspond to its molecular weight measured by the markers that assume full polypeptide unfolding, a special set of standards for appropriate globule sizes is necessary to evaluate the oligomeric state. In this paper, we used recombinant GFP and DsRed2 (Clontech, Mountain View, CA) proteins as monomeric and tetrameric standards, respectively. In addition to SDS-PAGE of unboiled samples with band visualization via native fluorescence, we also ran the same samples after boiling (i.e., under fully denaturing conditions) on the same gels, and used coomassie staining to identify the bands specific for the unboiled samples.

### Site-directed mutagenesis

To reduce aggregation of pmimGFP1, several amino acid changes in its N terminus were introduced by re-amplifying the full coding sequences with modified primers originally designed to amplify the inserts for the bacterial expression constructs. The introduced mutations were: K5E (mutant 1), K5T (mutant 2), and K5T, C8S, R9A (mutant 3).

### pH stability

Chromophore sensitivities to changes in pH were assayed for pmimGFP1, pmimGFP2, pmimGFP1 (K5T, C8S, R9A), and EGFP (BioVision, Mountain View, CA). Roughly 10 µg of the proteins (5μg for EGFP) were incubated in buffers of varying pH for 10 min at 25°C, followed by measuring the maximum fluorescence intensity of each. All the proteins were excited at 450 nm and emission was measured from 480 to 600 nm. The buffers included: 0.1 M glycine/HCl (pHs 3.0 and 3.5), 0.1 M sodium acetate (pHs 4.0, 4.5 and 5.0), 0.1 M phosphate (pH 6.0), 0.1 M HEPES (pH 7.0), 0.1 M Tris/HCl (pHs 8.0 and 9.0), 0.1 M carbonate (pHs 10.0 and 11.0), 0.1 M phosphate/NaOH (pHs 11.5, 12.0, 12.5, and 13.0) and 0.1 M NaOH (pH 13.5). Data were collected and graphed using the same instruments as for brightness measurements.

### Photostability

1μl of protein solutions - EGFP (BioVision, Mountain View, CA), pmimGFP1, and pmimGFP1 (K5T, C8S, R9A) - at approximately 1 µg/ml concentration were added to 100μl of immersion oil (Fluka/Sigma, St. Louis, MO), and vortexed for 5 s to obtain emulsion. To generate negative control droplets, 1μl of 1X PBS was emulsified in the same way and mixed in equal proportions with the protein emulsions. A droplet of this combined emulsion was placed onto a slide and slip-covered, with 3 replicate slides made for each protein. Individual droplets on the slides were illuminated through a 40× objective (Eclipse E600 microscope, Super High Pressure Mercury Lamp, CFI PLAN APO 40× objective, FITC-HYQ filter, Nikon, Japan) over the course of 10 minutes while collecting images every 30 s (exposure 800 ms, TV Lens C-0.6x, Nikon, OpenLab Software by Improvision, UK). The integrated density (sum of all pixel values) of a non-fluorescent droplet (filled with 1X PBS) was used as a background and subtracted from the density of a corresponding fluorescent droplet (on the same slide), with the help of Image J software (National Institutes of Health, Behthesda, MD). These values were plotted against time, and half-time of bleaching for the newly cloned proteins was inferred relative to EGFP.

## Results

### Sequence and phylogenetic analysis

The amino acid sequences of the two isoforms of *P. mimocerami* GFPs are 97% identical (only 6 amino acids difference). In the nucleotide-based phylogenetic tree of pontellid GFPs they appear as sister taxa (Fig. 1C). Overall, 37% of the amino acid sequence is identical among all of the copepod GFP-like sequences.

### Spectroscopic characteristics of pmimGFP1 and pmimGFP2

Both of the purified *Pontella* GFPs were soluble in PBS with 500 mM imidazole during the final elution step of purification. However, when the imidazole was removed, the proteins tended to eventually form large aggregates that almost completely precipitated out of solution. In order to perform the spectroscopic analysis, we added 3 M imidazole to the protein solutions to a final concentration of 250 mM, which re-solubilized the aggregates. EGFP protein, which served as a quantum yield standard, was assayed in parallel under identical conditions.

The absorption and emission spectra of pmimGFP1 and pmimGFP2 are identical (Fig. 2A), peaking at 491 nm and 505 nm, respectively. They are very similar to other copepod GFP-like proteins, which have absorbance max between 480 nm–490 nm and emission max between 500 nm–511 nm [10], [13]. The proteins possess identical molar extinction coefficients and quantum yields, which is not surprising given their high sequence similarity. Both their molar extinction and quantum yields are considerably higher than of EGFP, as measured in a direct comparison (Fig. 2, B and C). The molar extinction coefficient of the new proteins is 79,000 M^−1^ cm^−1^, lower than the average copepod molar extinction coefficient of about 89,000 M^−1^ cm^−1^.Assuming the quantum yield of EGFP QY = 0.6 [22], the quantum yield of the new proteins amounts to 0.92, approaching the theoretical maximum of 1 and notably exceeding even the highest value seen in other copepod GFP-like proteins (GFP from *Pontella meadi*, QY = 0.74, [10]).

### Oligomeric status of pmimGFP1 and pmimGFP2

**Figure 3:**
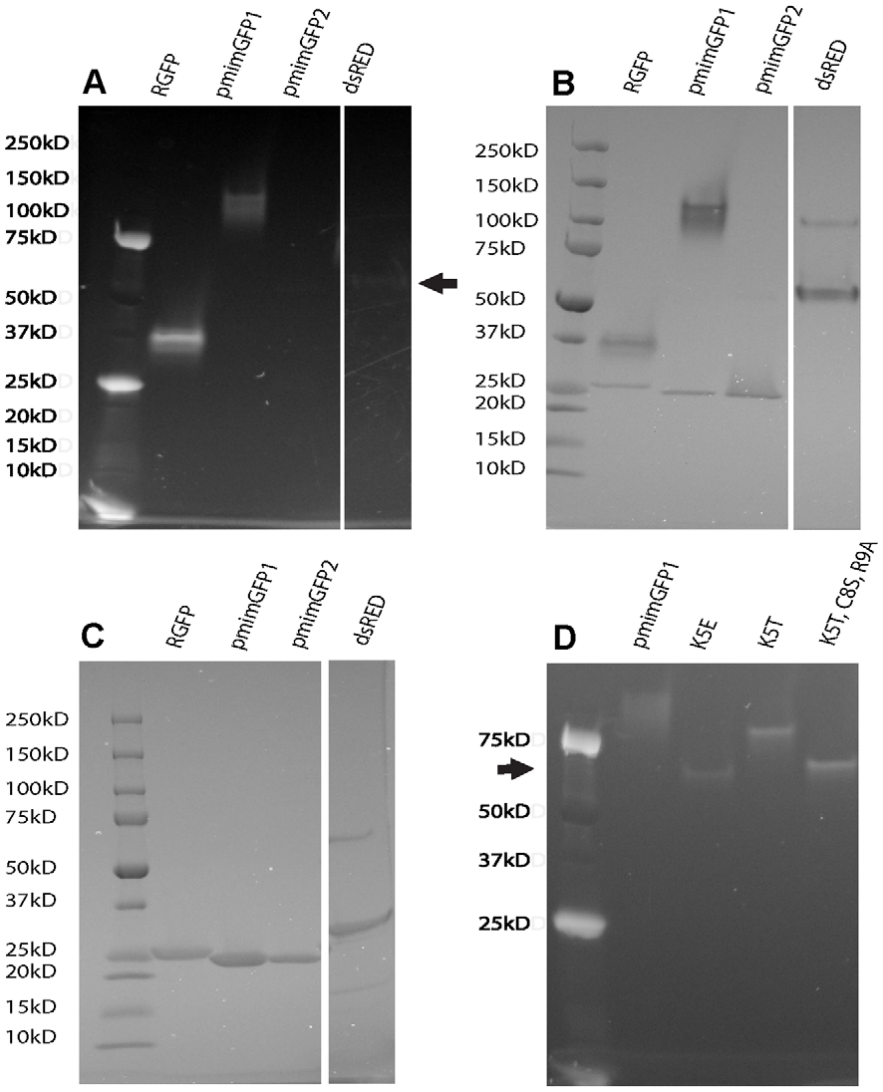
Aggregation and oligomerization analysis of the recombinant proteins. **A**. Recombinant fluorescent proteins electrophoresed on a SDS-containing gel without prior heating and viewed with UV illumination. pmimGFP1 fluoresces brightly, pmimGFP2 is also visible, but is very faint. rGFP is a monomer, DsRed is a tetramer (indicated by an arrow). B. The same gel as in figure 3A, but imaged with Coomassie stain. rGFP, pmimGFP1, and pmimGFP 2 are all susceptible to partial denaturation under the running conditions and therefore show non-fluorescent bands at ∼25 kD, the expected size of a fully denatured protein. DsRed, the tetrameric standard, retains its multimeric state. C. The same samples as in figures A and B, but electrophoresed in fully denaturing conditions (before loading, the samples were boiled 5 min) followed by Coomassie stain. All four samples show the single major band corresponding to the denatured protein at about 25 kD. D. Removal of N-terminal positive charges reduces aggregation. Lane 1 is the wild type pmimGFP1, lane 2 K5E mutant, lane 3 is K5T mutant, and lane 4 is a triple mutant K5T, C8S, and R9A. Arrow indicates tetramer mobility.

SDS-PAGE of unboiled samples of pmimGFP1 and pmimGFP2 shows native fluorescence as lower mobility bands as compared to the monomeric recombinant GFP (rGFP) and even tetrameric DsRed proteins (Fig. 3A–C), which suggests aggregated of high-order oligomeric forms. There seems to be a pronounced difference between the resistances of pmimGFP1 and pmimGFP2 to SDS-induced unfolding. In SDS-PAGE of unboiled samples, pmimGFP1 fluoresces strongly, while pmimGFP2 is barely visible roughly at the tetramer mobility (Fig. 3A). Coomassie staining of the same lanes (Fig. 3B) indicated that the majority of pmimGFP2 protein appears as a band at 25 kDa, corresponding to the mobility of the protein under fully denatured conditions (note that every GFP-like protein in this gel actually unfolds somewhat in SDS even without boiling). Figure 3C shows all of the proteins in a fully denatured state, with all protein masses around 25 kDa. Both copepod proteins are 222 amino acids long with a predicted molecular weight of 25 kDa.

### Mutations in the N-terminus to alleviate aggregation

A previous study demonstrated that, in many GFP-like proteins, the aggregation tendency can be reduced by replacing a few positively charged amino acids in the N-terminus by neutral or negatively charged ones [24]. We chose to replace three amino acids, two positively charged ones (K5 and R9), and one cysteine (C8) as a potential disulphide bridge-forming one. Figure 3D shows an SDS-PAGE of unboiled samples of mutants of pmimGFP1. Mutant 1 (K5E) shows increased mobility (i.e., less aggregation/oligomerization), but also substantially decreased brightness. Mutant 2 (K5T) is still very bright, but shows no change in mobility. Mutant 3 (K5T, C8S, R9A) matches the mobility of our tetrameric standard (DsRed2) and appears bright in the gel. We conclude that, although the mutagenesis alleviates aggregation, our best mutant protein still forms oligomers, most likely tetramers. Despite its apparent brightness, the quantum yield of the triple mutant is considerably lower (0.36) than in the parent protein (Fig. 2C), indicating that either the breakdown of the higher-order aggregates, or the effect of the particular mutations within a single monomer, was detrimental for the protein's brightness characteristics. We didn't evaluate the ME of the triple mutant because it was created without a six-histidine tag and therefore could not purify it from the crude bacterial lysate to measure the ME based on protein concentration assay.

### pH- and photostability

**Figure 4:**
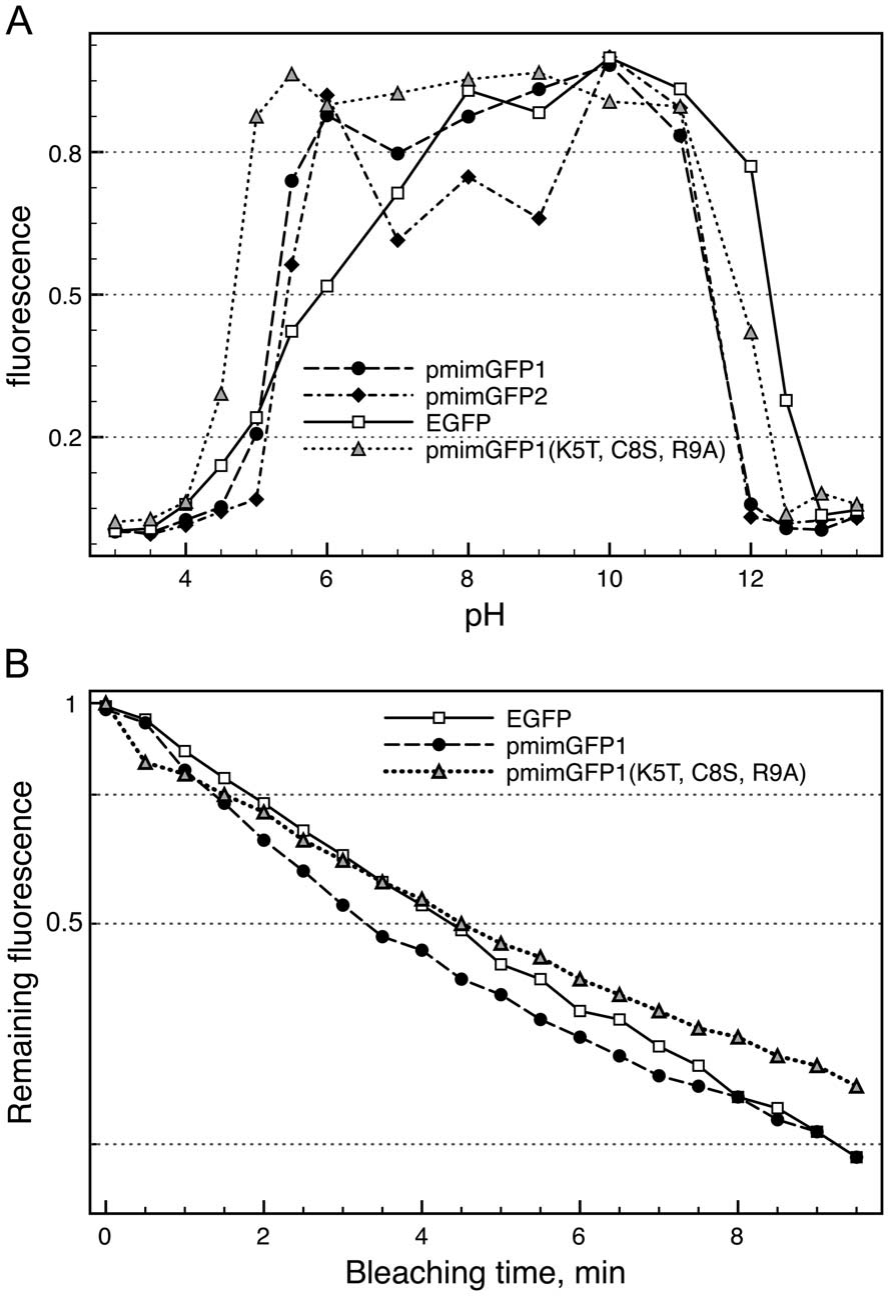
pH (A) and photostability (B) of novel copepod proteins and of the non-aggregating mutant of pmimGFP1. On both panels, analogous measurements of commercially available recombinant EGFP protein are presented as a reference. On panel B, the horizontal axis is time of illumination under the fluorescence microscope, and the scale of the vertical axis is logarithmic. On both panels, each point represents an average of three replicate measurements.

The new proteins are more stable in acidic pH than EGFP, demonstrating a pKa around 5.3–5.4, with the non-aggregating mutant of pmimGFP1 (K5T, C8S, R9A) being the most stable across the whole pH range, with a pKa of 4.7 (Fig. 4A). pmimGFP2 also exhibits a tendency to be less bright in the neutral pH range, which, however, is not always reproducible and may depend on other factors such as protein concentration and temperature fluctuations. Photostability was assayed for pmimGFP1 and its non-aggregating mutant relative to EGFP in the conditions approximating a typical application of the protein as a genetically encoded fluorescent label, by comparing the rates of photobleaching of protein emulsion droplets under the fluorescent microscope (Fig. 4B). The time to half-photobleaching of pmimGFP1 is 0.8 of EGFP, while its non-aggregating mutant is essentially identical to EGFP in this regard. Both pmimGFP1 and its mutant show non-exponential kinetics of photobleaching, with the highest photobleaching rate at the start of exposure. Notably, past the half-bleaching point both proteins photobleach at a similar rate, which is slightly lower than for EGFP (Fig. 4B).

## Discussion

Copepod luminescence was first documented long ago [26], and it was also observed that some luminescent species exhibited an additional fluorescence located at the site of the luminous glands [27]. However, the genus of copepods that we collected, *Pontella*, exhibits only green fluorescence and no luminescence [28]. Although copepods don't feature compound eyes such as some other crustaceans, the Pontellidae median eye is well developed, featuring an elaborate triple-lens construction in the ventral eyes of males [28], [29]. It has been previously suggested that green fluorescence may serve as a mate recognition/attraction signal in these copepods by creating a contrast with the blue background of the oceanic water [10]. It is also tempting to speculate that the very bright whole-body green fluorescence such as in *P. mimocerami* (Fig. 1B) may serve as a counter-shading mechanism under some ecologically relevant situations. This function would be analogous to the well-documented function of bioluminescence in dim ocean zones [10], [29], [30], [31]. Detailed modeling of fluorescence-driven light field transformations and their visual effect are required to substantiate this tentative suggestion, which is beyond the scope of this paper.

Figure 1 C shows the phylogenetic tree of all of the known copepod GFP-like proteins based on their respective nucleotide coding sequences. The tree suggests that the two GFP isoforms that we isolated represent a very recent gene duplication, which is in line with the noted abundance of closely related GFP genes in sequenced genomes. One previous observation that best highlights the continuous process of GFP gene duplication is that in lancelets (genus *Branchiostoma*) there are GFP gene copies specific to individual species within the genus [12].

The light transforming chromophores of both pmimGFP1 and pmimGFP2 proteins contain the same amino acid sequence, Gly-Tyr-Gly, as the other known copepod GFP-like proteins; the Tyr and second Gly are strictly conserved among all FPs. Also, the Arg and Glu amino acids responsible for the autocatalytic steps of chromophore formation are present at positions 96 and 222, respectively, according to GFP numbering (positions 87 and 221 in the pmimGFPs).

Although the first GFP-like proteins from copepods were reported as monomeric, it has since been established that they form tetramers [10], [31]. Our data suggest that native pmimGFP1 forms tetramers or aggregates of higher order (Fig. 3A–C), which is very common for natural fluorescent proteins [23], [32], [33]. pmimGFP2, despite very high sequence similarity to pmimGFP1, seems to be much more sensitive to the presence of SDS: it almost completely unfolds even when the sample is not heated, with the remaining native protein running as a very faint band roughly corresponding to the tetrameric size (Fig. 3A, B). The instability of pmimGFP2 under our native electrophoresis conditions prevents us from drawing conclusions about its oligomerization or aggregation tendency relative to pmimGFP1.

When purified, both pmimGFP1 and pmimGFP2 aggregate and, with time, almost completely precipitate out of solution. ppluGFP2, another copepod GFP-like protein, has a similar tendency to aggregate [10]. It was suggested this aggregation may be the result of electrostatic interactions between the charged surfaces of the fluorescent protein [31]. A site-directed mutagenesis approach developed for anthozoan GFP-like proteins [24] was applied to the new pmimGFPs to reduce aggregation. We replaced several amino acid residues (K5, C8, and R9) at the N terminus with other amino acids (E, T, S, or A) that are less likely to facilitate aggregation. Our third mutant, containing all these changes, was the most successful since it did not show aggregation beyond the tetrameric level and appeared bright in the expressing bacterial cells as well as on the polyacrylamide gel (Fig. 3). This non-aggregating mutant also demonstrated higher pH stability (pKa = 4.7, Fig. 4A) and photostability (Fig. 4B) than its ancestral pmimGFP1. Unfortunately, its quantum yield turned out to be quite low (0.36, Fig. 2C), despite its bright appearance.

The brightness of a GFP-like protein is proportional to the product of two factors: molar extinction coefficient (ME) and quantum yield (QY). In a direct comparison of the brightness characteristics between the new proteins and EGFP [22], which is the most widely used genetically encoded fluorescent marker and a typical reference point for brightness comparisons, the new proteins turned out to be 2.2-fold brighter overall, since both their ME and QY are higher (Fig. 2B, C). Remarkably, this makes them brighter than any FP currently in use in biotechnology [34], barring the possibility of inaccurate (lower than actual) brightness measurements in the previously reported FPs. Thus, these new copepod GFP-like proteins have a potential to become excellent reporters, at least in applications that tolerate oligomeric FP labels (such as monitoring promoter activity, organelle tracking, or cell and tissue labeling). Extensive mutagenesis would still be required to adapt these new proteins for imaging applications involving molecular fusions, which must rely on monomeric protein tags. It remains to be seen whether the natural tendency of the new proteins to aggregate and oligomerize can be alleviated by mutagenesis without compromising their exceptional brightness.
